# Perioperative and short-term results of the surgical treatment of infective endocarditis

**DOI:** 10.1186/1753-6561-6-S4-P5

**Published:** 2012-07-09

**Authors:** SL Moşteoru, LM Lucuta, M Gaspar, H Feier

**Affiliations:** 1 “Victor Babeş” University of Medicine and Pharmacy, Timişoara, Romania; 2 Timişoara Institute of Cardiovascular Medicine, IInd Cardiovascular Surgery Department, Romania

## Introduction

Infective endocarditis is defined as the infection of the endocardial surface of the heart, which may include one or more heart valves or the mural endocardium. It results in severe valvular insufficiency, which may lead to congestive heart failure, and myocardial abscesses. One of the most feared complications is thromboembolism, through infected emboli. The purpose of this paper is to assess the perioperative and short term results of the IInd Cardiovascular Surgery Department in the treatment of infective endocarditis during a three year period, from January the first, 2002 to September the first, 2005.

## Methods

We conducted a study on 31 patients operated upon during this timeframe. They represent 0.81% of the total number of 3795 surgical procedures performed in our clinic. 83.87% were male, with a mean age of 47.41 years. Eighteen (18/31, 58.06 %) were acute cases, out of which 29,03% (9/31) had the pathogenic agent identified by hemocultures and culture of the explanted valves. Three patients presented preoperative peripheral embolic events (9.67%). The aortic valve was affected in 74.19% cases (23/31), the mitral valve in 41.93% (13/31) whereas there was a single tricuspid valve lesion (3.22%, 1/31). Three of these cases were redo procedures (9.67%).

## Results

There were 35 valve replacements performed, 2 associated tricuspid plasties and two patients benefited from associated procedures: triple coronary artery bypass grafting and ascending aortic replacement. One patient presented a false aneurysm of the aortic root. The prosthesis used were mechanical (23/31, 74.19%), biological (6/31, 19.35%), homograft (1/31, 3.22%) or autograft (1/31, 3.22%). The operative (<30 days) mortality was 3.22% (a single patient expired). The mean follow up period was 15.708.54 months. Global survival at 1,6 and 12 months was 95.654.25%.

## Conclusions

Infective endocarditis is a severe and potentially lethal disease but if a proper management is observed, there is a strong determinant for survival as proved by our study where the surgical treatment offers a short term survival which is not different from the one in the general population.

